# Human Parasitism by *Amblyomma parkeri* Ticks Infected with *Candidatus* Rickettsia paranaensis, Brazil

**DOI:** 10.3201/eid2512.190988

**Published:** 2019-12

**Authors:** Ana Beatriz P. Borsoi, Karla Bitencourth, Stefan V. de Oliveira, Marinete Amorim, Gilberto S. Gazêta

**Affiliations:** Instituto Oswaldo Cruz, Rio de Janeiro, Brazil (A.B.P. Borsoi, K. Bitencourth, M. Amorim, G.S. Gazêta);; Universidade Federal de Uberlândia, Uberlândia, Brazil (S.V. de Oliveira);; Ministério da Saúde do Brasil, Brasília, Brazil (S.V. de Oliveira)

**Keywords:** human parasitism, Amblyomma parkeri, Candidatus Rickettsia paranaensis, ticks, vector-borne infections, rickettsia, bacteria, spotted fever, zoonoses, Atlantic rainforest, Brazil

## Abstract

Spotted fever is the main rickettsial disease in Brazil. We report 12 cases of human parasitism by *Amblyomma parkeri* in the Atlantic rainforest, an area of Brazil to which spotted fever is endemic. Nine of the ticks were infected with *Candidatus* Rickettsia paranaensis.

Spotted fever is considered the main tickborne disease in South America (*1*). In Brazil, spotted fever has been reported since the 1920s and is known to show great clinical diversity and ecoepidemiologic scenario complexity, involving *Rickettsia rickettsii* transmitted by *Amblyomma sculptum* and *A. aureolatum* ticks and *Rickettsia parkeri* strain Atlantic rainforest vectored by *A. ovale* ticks (*2*). However, several studies have identified different *Rickettsia* species infecting a variety of tick species in Brazil, indicating the possibility of newly emerging spotted fever scenarios in Brazil (*1**–**3*).

In southern Brazil, in addition to the scenario already established for the Atlantic forest region, studies indicate the possibility of a unique cycle developing in the Pampa biome, in which *R. parkeri* sensu stricto might be associated with spotted fever cases involving an *A. tigrinum* tick vector (*3*). Accordingly, to expand the understanding of the spotted fever scenario in Brazil, we conducted a molecular study of *Rickettsia* in *A. parkeri* ticks as parasites of humans in an area of Brazil to which spotted fever is endemic.

During 2013–2018, in an investigation and surveillance of spotted fever cases in urban areas near Atlantic rainforest fragments in the Parana, Santa Catarina, and Rio Grande do Sul states in southern Brazil, we collected 12 tick nymphs parasitizing humans and morphologically identified these ticks as *A. parkeri* (*4*). We individually processed 11 specimens for DNA extraction (*5*), subjected this DNA to PCR for molecular confirmation of tick species (*6*), and isolated *glt*A, *htr*A, *omp*A, and *omp*B gene fragments (Appendix Table). We purified PCR products, sequenced them, and compared them with rickettsial sequences available in GenBank. We subjected concatenated aligned rickettsial sequences to maximum-likelihood analysis.

We identified *A. parkeri* ticks with containing rickettsia in all 3 states studied. Nine samples amplified fragments from >1 of the 4 rickettsia gene markers studied. All sequences for *omp*B and *omp*A gene fragments showed 100% similarity with *Candidatus* Rickettsia paranaensis (GenBank accession nos. KX018050, JN126322, and JN126321). The *htr*A and *glt*A sequences had 100% similarity to many of the spotted fever group rickettsia, including *Candidatus* R. paranaensis (GenBank accession nos. KX018052 and JN126320). Phylogenetic analysis showed that bacteria detected in *A. parkeri* ticks from southern Brazil were in the same clade as *Candidatus* R. paranaensis (Figure).

**Figure:**
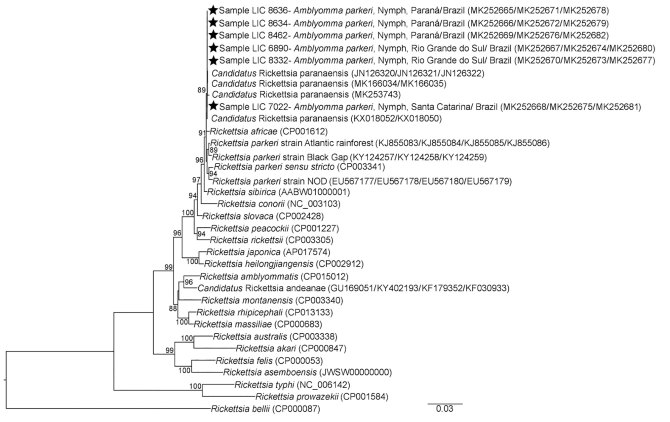
Concatenated phylogenetic analysis of rickettsia gene fragments detected in *Amblyomma parkeri* ticks in Brazil. Gene fragments *glt*A (1,013 bp), *htr*A (370 bp), *omp*A (494 bp), and *omp*B (822 bp) were inferred by maximum-likelihood analysis with the evolution model T92 + G (Tamura model). Values on the branches indicate bootstrap values (cutoff value 70%). Stars indicate sequences obtained in this study. GenBank accession numbers are given in parentheses. Scale bar indicates nucleotide substitutions per site.

The pathogenicity of *Candidatus* R. paranaensis is unknown. However, Peckle et al. (*7*) placed it close to the Old World species *R. africae* and *R. sibirica*, both of which are proven pathogenic species (*1*). *A. parkeri* nymphs infected by *Candidatus* R. paranaensis are not uncommon (*7*) and might have high frequencies of infection. Luz et al. (*8*) reported that 75% of passariform birds in southeastern Brazil were infected with ticks, a value similar to that obtained in this study (81.81%) for humans in the southern region. Thus, circulation of *Candidatus* R. paranaensis in the Atlantic Forest biome might be closely associated with the presence of *A. parkeri* immature tick stages and passeriform birds.

Although reports of human parasitism by tick species of the genus *Amblyomma* are increasing, *A. parkeri* ticks have been rarely reported from humans, although there are reports of parasitism in the Atlantic rainforest area of southeastern Brazil, including a high prevalence of this ixodid (nymphs) on humans in Rio Grande do Sul State (*9*,*10*). Although these reports were for a region to which spotted fever is endemic, there was no study of the associated rickettsia. However, our results show 12 humans parasitized by *A. parkeri* nymphs in the 3 states that comprise the southern region of Brazil, indicating that the parasitism of humans by such ticks is more common than that reported. Examples of *Candidatus* R. paranaensis in *A. parkeri* parasitizing humans in an area to which spotted fever is endemic, with milder clinical characteristics (*2*), highlight the need to investigate the role of vector and rickettsia in spotted fever in southern Brazil. This investigation should help in formulating appropriate public health responses by existing surveillance programs.

Appendix Additional information on human parasitism by *Amblyomma parkeri* ticks infected with *Candidatus* Rickettsia paranaensis, Brazil.
