# Supplementary material for: Human Parasitism by Amblyomma parkeri Ticks Infected with Candidatus Rickettsia paranaensis, Brazil
Source: Emerg Infect Dis. 2019 Dec;25(12):2339–41. doi: 10.3201/eid2512.190988 (PMC6874247; doi:10.3201/eid2512.190988)
Supplement: Appendix — Additional information on human parasitism by Amblyomma parkeri ticks infected with Candidatus Rickettsia paranaensis, Brazil. [file 19-0988-Techapp-s1.pdf]

Article DOI: https://doi.org/10.3201/eid2512.190988

Human Parasitism by *Amblyomma parkeri* Ticks Infected with *Candidatus* Rickettsia paranaensis, Brazil

Appendix

Appendix Table. Primers used for PCRs to study human parasitism by *Amblyomma parkeri* ticks infected with *Candidatus* Rickettsia paranaensis, Brazil*

Gene	PCR characteristic	Primer	Nucleotide sequence, 5'→3'	Fragment, bp	Reference†
*glt*A	NA	CS2-78	GCAAGTATCGGTGAGGATGTAAT	401	(*1*)
	NA	CS2-323	GCTTCCTTAAAATTCAATAAATCAGGAT		
	NA	CS4-239	GCTCTTCTCATCCTATGGCTATTAT	834	(*2*)
	NA	CS4-1069	CAGGGTCTTCGTGCATTTCTT		
*htr*A	Nested, primary round	17k-5	GCTTTACAAAATTCTAAAAACCATATA	549	(*2*)
	Nested, primary round	17k-3	TGTCTATCAATTCACAACTTGCC		
	Secondary round	17Kd1	GCTCTTGCAACTTCTATGTT	434	(*3*)
	Secondary round	17Kd2	CATTGTTCGTCAGGTTGGCG		
omp B	Nested, primary round	ompB-OF	GTAACCGGAAGTAATCGTTTCGTAA	511	(*4*)
	Nested, primary round	ompB-OR	CTTTATAACCAGCTAAACCACC		
	Secondary round	ompB SFG-IF	GTTTAATACGTGCTGCTAACCAA	425	(*4*)
	Secondary round	ompB SFG/TG-IR	GGTTTGGCCCATATACCATAAG		
	NA	120-M59	CCGCAGGGTTGGTAACTGC	862	(*5*)
	NA	120-807	CCTTTTAGATTACCGCCTAA		
omp A	NA	Rr 190.70p	ATGGCGAATATTTCTCCAAAA	532	(*6*)
	NA	Rr 190.602n	AGTGCAGCATTCGCTCCCCCT		

*NA, not applicable.
†References for oligonucleotides and respective amplification protocols used.

References

1. Labruna MB, Whitworth T, Horta MC, Bouyer DH, McBride JW, Pinter A, et al. *Rickettsia* species infecting *Amblyomma cooperi* ticks from an area in the state of São Paulo, Brazil, where Brazilian spotted fever is endemic. J Clin Microbiol. 2004;42:90–8. PubMed https://doi.org/10.1128/JCM.42.1.90-98.2004

2. Labruna MB, McBride JW, Bouyer DH, Camargo LM, Camargo EP, Walker DH. Molecular evidence for a spotted fever group *Rickettsia* species in the tick *Amblyomma longirostre* in Brazil. J Med Entomol. 2004;41:533–7. PubMed

3. Webb L, Carl M, Malloy DC, Dasch GA, Azad AF. Detection of murine typhus infection in fleas by using the polymerase chain reaction. J Clin Microbiol. 1990;28:530–4. PubMed

4. Choi YJ, Jang WJ, Ryu JS, Lee SH, Park KH, Paik HS, et al. Spotted fever group and typhus group rickettsioses in humans, South Korea. Emerg Infect Dis. 2005;11:237–44. PubMed https://doi.org/10.3201/eid1102.040603

5. Roux V, Raoult D. Phylogenetic analysis of members of the genus *Rickettsia* using the gene encoding the outer-membrane protein rOmpB (*ompB*). Int J Syst Evol Microbiol. 2000;50:1449–55. PubMed https://doi.org/10.1099/00207713-50-4-1449

6. Regnery RL, Spruill CL, Plikaytis BD. Genotypic identification of rickettsiae and estimation of intraspecies sequence divergence for portions of two rickettsial genes. J Bacteriol. 1991;173:1576–89. PubMed https://doi.org/10.1128/jb.173.5.1576-1589.1991
